# Dronedarone versus sotalol in patients with atrial fibrillation: A systematic literature review and network meta‐analysis

**DOI:** 10.1002/clc.24011

**Published:** 2023-04-06

**Authors:** Jagmeet P. Singh, Carina Blomström‐Lundqvist, Mintu P. Turakhia, A. John Camm, Mir Sohail Fazeli, Bahij Kreidieh, Christopher Crotty, Peter R. Kowey

**Affiliations:** ^1^ Cardiology Division, Harvard Medical School Massachusetts General Hospital Boston Massachusetts USA; ^2^ Department of Cardiology, School of Medical Sciences, Faculty of Medicine and Health Örebro University Örebro Sweden; ^3^ Department of Medical Science Uppsala University Uppsala Sweden; ^4^ Department of Medicine (Cardiovascular Medicine), Center for Digital Health Stanford University Stanford California USA; ^5^ Cardiac Academic Group St. George's University of London London UK; ^6^ Evidinno Outcomes Research Inc. Vancouver Canada; ^7^ The Lankenau Institute for Medical Research Wynnewood Pennsylvania USA; ^8^ Department of Medicine Thomas Jefferson University Philadelphia Pennsylvania USA

**Keywords:** antiarrhythmia agents, atrial fibrillation, dronedarone, network meta‐analysis, sotalol, systematic review

## Abstract

**Background:**

There are limited comparative data on safety and efficacy within commonly used Vaughan‐Williams (VW) class III antiarrhythmic drugs (AADs) for maintenance of sinus rhythm in adults with atrial fibrillation (AF).

**Hypothesis:**

We hypothesized that dronedarone and sotalol, two commonly prescribed VW class III AADs with class II properties, have different safety and efficacy effects in patients with nonpermanent AF.

**Methods:**

A systematic literature review was conducted searching MEDLINE®, Embase, and Cochrane Central Register of Controlled Trials (CENTRAL) up to June 15, 2021 (NCT05279833). Clinical trials and observational studies that evaluated safety and efficacy of dronedarone or sotalol in adults with AF were included. Bayesian random‐effects network meta‐analysis (NMA) was used to quantify comparative safety and efficacy. Where feasible, we performed sensitivity analyses by including only randomized controlled trials (RCTs).

**Results:**

Of 3581 records identified through database searches, 37 unique studies (23 RCTs, 13 observational studies, and 1 nonrandomized trial) were included in the NMA. Dronedarone was associated with a statistically significantly lower risk of all‐cause death versus sotalol (hazard ratio [HR] = 0.38 [95% credible interval, CrI: 0.19, 0.74]). The association was numerically similar in the sensitivity analysis (HR = 0.46 [95% CrI: 0.21, 1.02]). AF recurrence and cardiovascular death results were not significantly different between dronedarone and sotalol in all‐studies and sensitivity analyses.

**Conclusion:**

The NMA findings indicate that, across all clinical trials and observational studies included, dronedarone compared with sotalol was associated with a lower risk of all‐cause death, but with no difference in AF recurrence.

## Graphical abstract

A systematic literature review and network meta‐analysis was conducted to assess the comparative safety and efficacy of dronedarone versus sotalol, two commonly used Vaughan‐Williams class III antiarrhythmic drugs, for maintenance of sinus rhythm in adults with atrial fibrillation (AF). Across all clinical trials and observational studies analyzed, dronedarone was associated with a lower risk of all‐cause death versus sotalol. Cardiovascular death and recurrence of AF were not significantly different between dronedarone and sotalol.

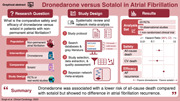

## 1 INTRODUCTION

Atrial fibrillation (AF) is the most common sustained cardiac arrhythmia in clinical practice and remains one of the major causes of stroke, transient ischemic attack, and heart failure.^1^, ^2^ Despite improvements in rhythm control interventions, there is still a considerable risk of mortality, AF‐related stroke, frequent hospitalization, and complications due to hemodynamic abnormalities and thromboembolic events in patients with AF.

The main antiarrhythmic drugs (AADs) for rhythm control and prevention of AF recurrence include Vaughan‐Williams class III drugs amiodarone, dofetilide, dronedarone, sotalol, and class Ic drugs flecainide and propafenone.^3^, ^4^ Reservations exist about the use of amiodarone as a long‐term therapy option due to well‐recognized extracardiac side effects.^3^, ^5^ Similarly, dofetilide has extensive contraindications due to a relatively high risk of torsades de pointes.^6^ Dronedarone and sotalol are both class III agents (with antiadrenergic effects) which have demonstrated similar efficacy,^7^, ^8^ and although current guideline and label recommendations emphasizing safety considerations vary for the two agents according to clinical circumstances of recurrent AF patient populations,^3^ United States (US) and European Union (EU) physician adherence to guideline recommendations for these and other AADs was shown in a recent study to be suboptimal.^9^ Direct comparisons between dronedarone and sotalol are limited, however, some evidence has shown that sotalol use is associated with a higher risk of ventricular proarrhythmia and increased overall mortality, recently recognized in the 2020 European Society of Cardiology (ESC) AF guidelines.^3^, ^7^, ^8^, ^10^

This systematic literature review (SLR) was undertaken to evaluate the evidence on the comparative safety and efficacy of two relatively similar treatments, dronedarone and sotalol, in adults with AF, from clinical trials and observational studies, and to combine and quantify these outcomes via network meta‐analysis (NMA).

## 2 METHODS

This SLR was carried out according to standard methods as per the Cochrane Handbook for Systematic Reviews of Interventions^11^ and the Preferred Reporting Items for Systematic Reviews and Meta‐Analyses (PRISMA) guideline.^12^ This review was registered with ClinicalTrials.gov (NCT05279833).

### 2.1 Data sources and search strategies

Searches were conducted in MEDLINE®, Embase, and CENTRAL (Cochrane Central Register of Controlled Trials) via OvidSP to capture records published up to June 15, 2021 (Supporting Information: Tables A.1–A.3). Additionally, conference abstracts published between 2019 and 2021 from the American College of Cardiology (ACC), American Heart Association (AHA), Heart Rhythm Society (HRS), and ESC congresses were searched, among others. Searches of US and EU clinical trial registry databases were also conducted to find articles that had reported results. Finally, “hand searches” of the reference lists of previously published literature reviews on the same topic were also conducted to capture additional eligible studies that were missed during the main database search.

### 2.2 Study selection

Study eligibility criteria for the SLR were defined using the PICO framework (Population, Intervention, Comparator, Outcome). Clinical trials and comparative observational studies that evaluated dronedarone and/or sotalol in adults with AF were included. Excluded were studies of patients with permanent AF.

Two independent senior reviewers were responsible for reviewing all abstracts according to the PICO criteria. Abstracts considered eligible for inclusion proceeded to a full‐text screening phase, where they were screened by the same reviewers. All records deemed eligible after full‐text screening were included in the SLR. At each stage of the screening process, any discrepancies between reviewers in the decision to include or exclude an article were resolved by a third reviewer to reach a consensus.

### 2.3 Data extraction and quality assessment

Data were extracted independently by two reviewers, and if discrepancies in interpretation could not be resolved, a third reviewer was consulted to reach consensus. Extraction included study characteristics, interventions, patient characteristics, as well as safety and efficacy outcomes. Baseline characteristics of interest were age, sex, race/ethnicity, comorbidities for AF, and mean left ventricular ejection fraction (LVEF). Safety outcomes were all‐cause death, cardiovascular (CV) death, ventricular proarrhythmia, and conduction disorders. Efficacy outcomes were CV hospitalization, AF hospitalization, heart failure hospitalization, stroke, myocardial infarction, and AF recurrence.

Two independent reviewers assessed the quality of the included studies using the Cochrane risk of bias tool for randomized trials^13^ and the Newcastle–Ottawa scale for observational studies.^14^ A third investigator intervened to reach consensus in case of unresolved conflicts between the decisions of the two reviewers.

### 2.4 Statistical analysis

Bayesian NMA was conducted to estimate the relative safety and efficacy of each treatment pair in the network for each outcome.^15^ The analysis was conducted using the RJAGS v4.1.2, BUGSnet v1.1, and forestplot v2.0.1 packages for R. Responses were modeled using a binomial distribution with either a log link (for dichotomous data) or a cloglog link (for survival data). In case of an absence of events in an arm of a study for a given outcome, 0.5 was added to each cell in the corresponding 2 × 2 table. Both fixed and random effects models were assessed; the random effects model was retained since it provided better fit as measured by the deviance information criterion. Models were fitted using Markov Chain Monte Carlo simulations implemented in JAGS version 4.3.0. Simulations were run for 30 000 iterations with three chains, a thinning rate of 1, and a burn‐in of 10 000. Convergence of the chains was ensured by visually inspecting trace and history plots.

The consistency assumption was assessed by (1) node splitting and (2) fitting a model which does not assume consistency and comparing its model fit to the traditional model. To address heterogeneity of study design, one analysis (all‐studies analysis) was conducted including observational studies, randomized controlled trials (RCTs), plus one nonrandomized clinical trial,^16^ and a sensitivity analysis was conducted restricted to RCTs (RCTs‐only analysis). In the all‐studies analysis, a normal–normal hierarchical model on study design was applied. Between‐study heterogeneity was assessed using the standard deviation across random effects models. Statistical significance was determined based on whether the 95% credible intervals (CrIs)—analogous to the 95% confidence interval in frequentist meta‐analysis—included the numeral one. Analyses of hazard ratios (HRs) were conducted where possible, while risk ratios (RRs) were analyzed when HRs were not available. For the RR analysis, no adjustment was made, and the latest available data were taken. For the HR analysis, log follow‐up time was included as a covariate in the analysis.

## 3 RESULTS

### 3.1 Study selection

**Figure 1:**
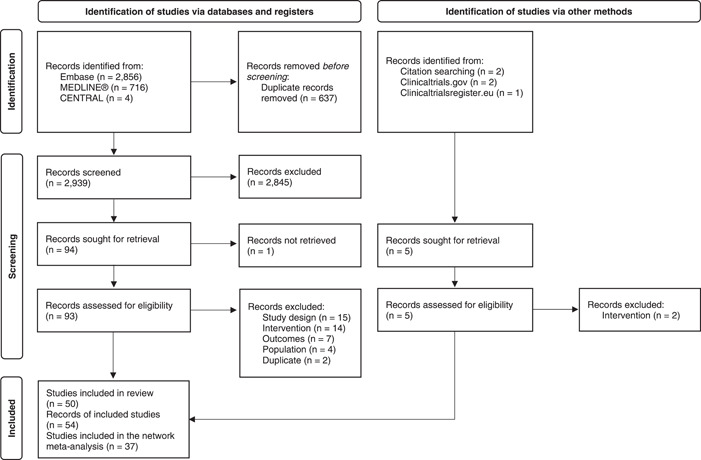
PRISMA flow diagram.

The search identified a total of 3576 records in MEDLINE®, Embase, and CENTRAL as well as five additional records from gray literature and hand searches. Following full‐text screening, 54 records pertaining to 50 unique studies were retained for qualitative synthesis as part of the SLR. Finally, 37 studies were judged to be sufficiently homogenous with outcomes data that was shared by at least one other study, and these were included in the NMA. The PRISMA diagram is presented in Figure 1. The full list of final included and excluded studies with reasons for exclusion at the full‐text review stage, as well as the corresponding publications per included trial, are presented in Supporting Information: Tables A.4 and A.5.

### 3.2 Study characteristics and interventions

The present SLR included a total of 50 studies: 27 RCTs, 19 retrospective cohort studies, 2 retrospective case‐control studies, 1 prospective cohort study, and 1 nonrandomized clinical trial (Figure 1). Sample sizes across included studies ranged from 33 patients to 312 341 patients (median = 404 patients). Among studies reporting arrhythmia‐related endpoints (*n* = 39), 26 described methods used for arrhythmia monitoring. Common methods for monitoring treatment efficacy included tele‐electrocardiogram recorders (*n* = 12), implantable devices (*n* = 3), 24‐h Holter monitoring (*n* = 2), or other unspecified ambulatory measurement devices (*n* = 3). Eight studies conducted rhythm measurements only at follow‐up visits. A summary of study characteristics is provided in Supporting Information: Table A.6.

Sotalol was administered in 37 studies, dronedarone and amiodarone were in 22 studies, and propafenone, flecainide, quinidine, and dofetilide in 14, 12, 7, and 4 studies, respectively. Sixteen studies were placebo‐controlled. A summary of intervention characteristics is available in Supporting Information: Table A.7.

### 3.3 Patient characteristics

The mean or median age of study participants ranged from 52^17^ to 76 years^18^ (overall median = 63 years; *k* [number of unique studies] = 46). The proportion of male patients ranged from 35%^19^ to 99.3%^20^ (median = 63.5%; *k* = 44). Among seven studies reporting race/ethnicity, the majority of patients were White (median = 88.0%).^20^, ^21^, ^22^, ^23^, ^24^, ^25^, ^26^ Comorbidities and mean LVEF were moderately‐ to well‐reported across included studies. A summary of patient characteristics at baseline is provided in Supporting Information: Table A.8.

### 3.4 Study quality assessment and risk of bias

The Cochrane risk of bias tool for randomized trials and the Newcastle–Ottawa scale for observational studies can be found in Supporting Information: Tables A.9 and A.10, respectively. In summary, the included studies were of generally high or moderate quality, indicated by low risk or unclear risk of bias in the Cochrane tool, and total scores of greater than 5 out of a possible 9 points for the Newcastle–Ottawa scale.

### 3.5 NMA

Of the 50 unique studies, the NMA comprised 37 studies that could be included in at least one network, consisting of 23 RCTs, 13 observational studies, and 1 nonrandomized clinical trial. Thirteen studies were excluded from the NMA due to: lack of analyzable outcomes (*k* = 7),^27^, ^28^, ^29^, ^30^, ^31^, ^32^, ^33^ having a comparator arm of “no AAD” or “other AAD,” where AADs of interest were mixed together (*k* = 4),^23^, ^24^, ^34^, ^35^ differences in the population compared with the rest of the evidence base (*k* = 1),^36^ and use of combination therapy (ranolazine and dronedarone vs. ranolazine alone; *k* = 1).^37^ Where data were available, sensitivity analyses were carried out with RCTs‐only. The full set of effect measures, number of studies, patients, and events is summarized in Supporting Information: Table A.11.

#### 3.5.1 Safety outcomes

**Figure 2:**
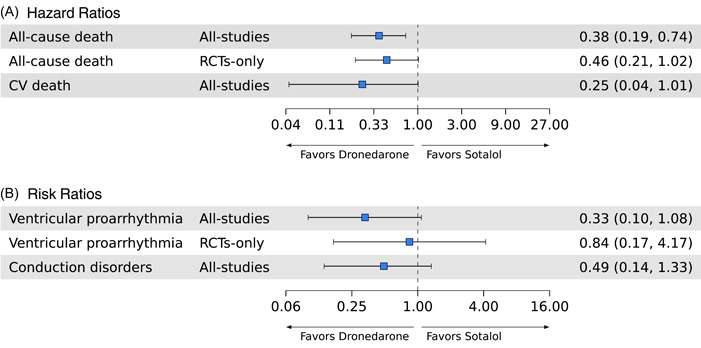
Network meta‐analysis of safety outcomes with dronedarone versus sotalol as (A) hazard ratios and (B) risk ratios. CV, cardiovascular; RCT, randomized controlled trial.

Risk of all‐cause death was statistically significantly lower for dronedarone versus sotalol (HR = 0.38 [95% CrI: 0.19, 0.74]; *k* = 22) in the analysis of all studies (Figure 2A). In the sensitivity analysis with RCTs‐only, risk of all‐cause death was still numerically lower with dronedarone, supporting the main analysis in terms of the magnitude and direction of effect size, despite the slightly wider 95% CrIs (HR = 0.46 [95% CrI: 0.21, 1.02]; *k* = 16). Risk of CV death was numerically lower for dronedarone compared with sotalol; however, the CrI did not indicate significance (HR = 0.25 [95% CrI: 0.04, 1.01]; *k* = 2).

For the all‐studies analysis, dronedarone was associated with a numerically lower risk of ventricular proarrhythmia relative to sotalol; however, this result did not reach significance (RR = 0.33 [95% CrI: 0.10, 1.08]; *k* = 23; Figure 2B). Sensitivity analysis with RCTs‐only also suggested a numerically lower risk of ventricular proarrhythmia with dronedarone, but results were not significant (RR = 0.84 [95% CrI: 0.17, 4.17]; *k* = 16). Risk of conduction disorders, including atrioventricular block or requirement for pacemaker implantation, was numerically lower with dronedarone versus sotalol (RR = 0.49 [95% CrI: 0.14, 1.33]; *k* = 4).

#### 3.5.2 Efficacy outcomes

**Figure 3:**
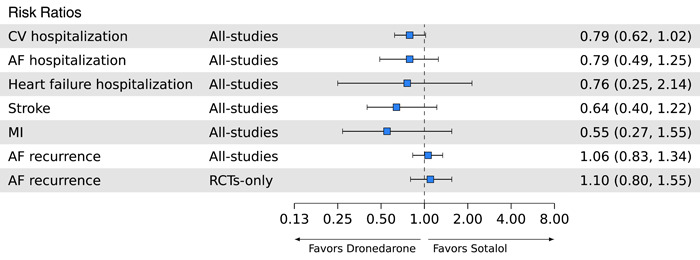
Network meta‐analysis of efficacy outcomes with dronedarone versus sotalol as risk ratios. AF, atrial fibrillation; CV, cardiovascular; MI, myocardial infarction; RCT, randomized controlled trial.

For risk of hospitalization due to heart failure (*k* = 4), AF (*k* = 2), or CV events (k = 4), RRs were consistent with numerically lower risk for dronedarone compared with sotalol—ranging from 0.76 to 0.79—however, in all cases, differences between treatments were not significant (Figure 3).

Risk of stroke (RR = 0.64 [95% CrI: 0.40, 1.22]; *k* = 6) and myocardial infarction (RR = 0.55 [95% CrI: 0.27, 1.55]; *k* = 2) were also numerically lower for dronedarone, although results did not reach significance.

Sensitivity analysis from RCTs‐only was available for AF recurrence, but not for other efficacy outcomes. There was no significant difference in risk of AF recurrence between dronedarone and sotalol in both the all‐studies (RR = 1.06 [95% CrI: 0.83, 1.34]; *k* = 17) and RCTs‐only (RR = 1.10 [95% CrI: 0.80, 1.55]; *k* = 14) analyses.

## 4 DISCUSSION

To our knowledge, this is the first SLR and NMA combining published data from clinical trials and observational studies to compare the safety and efficacy of dronedarone versus sotalol in AF, considering the benefit‐risk profile of the Vaughan‐Williams class III and class II properties of these two AADs. A major finding from this NMA included evidence for a lower risk of all‐cause death with dronedarone relative to sotalol. These findings are consistent with a previous NMA of RCTs by Freemantle et al.,^7^ as well as a more recent meta‐analysis of RCTs as part of a Cochrane Review by Valembois et al.^8^ In both of these meta‐analyses, effect measures for the selected drugs were estimated relative to placebo, with evidence of higher risk of death for sotalol, and lower risk of stroke with dronedarone. All 23 RCTs that were included in the present NMA were also included in the Cochrane Review.^8^ The inclusion of real‐world evidence (RWE) from observational studies with head‐to‐head comparison of treatments commonly used for maintenance of sinus rhythm in adults with nonpermanent AF in the current review represents a novel approach to evaluating treatment options in AF.

For all‐cause death, there were seven observational studies contributing to the analysis, published between 2009 and 2020.^10^, ^18^, ^25^, ^38^, ^39^, ^40^ One study from this set reporting outcomes from the Duke Databank for Cardiovascular Disease found that death rates were lower in patients treated with sotalol versus amiodarone or no AAD.^25^ Four of these observational studies comprised large national database data sets from Sweden,^10^, ^18^ Denmark,^38^ and South Korea.^39^ In the Danish study (2009), published at a time when sotalol, flecainide, and propafenone were guideline‐recommended first‐line treatments, no increased death rate was evident in patients treated with these AADs or amiodarone compared with no treatment.^38^ The more recent Swedish study (2018) reported lower all‐cause death with dronedarone or flecainide versus sotalol; however, this study also showed a potential for hidden confounding,^10^ whereas the earlier Swedish study (2014) reported that overall death rates with AF patients on dronedarone were lower than the general population.^18^ In the Korean study, no difference in the risk of death from any cause was evident between dronedarone and sotalol.^39^ In this NMA, risk of CV death was numerically lower with dronedarone by 75%, however, this result was based on only two studies,^39^, ^41^ and failed to reach statistical significance. It should be noted that the inclusion of RWE in this analysis of dronedarone versus sotalol provides a naturalistic means of comparing perhaps broadly similar AF patient populations, based on the proportions of specific AF patient populations in the US and EU receiving dronedarone or sotalol.^9^

Recent AF guideline updates have led to clear differences in first‐line treatment recommendations for these two medications in current clinical guidelines for AF patients with heart failure with reduced ejection fraction (HFrEF).^3^, ^4^ ESC guidelines recommend that sotalol should not be used,^3^ and AHA/ACC/HRS guidelines recommend excluding or using with caution in heart failure (except with implantable cardioverter defibrillator).^4^ The recently published Antiarrhythmic Medication for Atrial Fibrillation (AIM‐AF; 2022) survey highlighted that 12% of responding EU physicians and 25% of US physicians still selected sotalol as a first‐line treatment in patients with HFrEF.^9^ ESC guidelines advise dronedarone only be used in AF patients with mildly impaired but stable LVEF including heart failure with preserved ejection fraction (HFpEF), and AHA/ACC/HRS guidelines advise to exclude or use with caution in AF patients with heart failure. However, the AIM‐AF survey found that 8% of EU and US physicians selected dronedarone as a first‐line treatment in patients with AF and HFrEF.^9^ The AIM‐AF physician survey highlighted quantifiable deviations between guideline recommendations and real‐world practice in AF patients, and it is very likely that similar suboptimal adherence to guideline recommendations is reflected in the data set from observational studies included in this NMA.

By definition, AADs lower the risk of AF recurrence, with an approximately two to five times lower rate of recurrence via maintenance of sinus rhythm.^7^, ^8^ Meta‐analysis of RCT data indicates similar rates of AF recurrence for dronedarone compared with sotalol (with placebo as a common comparator), and both the all‐studies analysis and RCTs‐only NMA results in this study are consistent with the findings of previous studies, suggesting that introduction of RWE does not bias the relative effect of dronedarone versus sotalol in AF recurrence.

The downside of antiarrhythmic treatment, that is, the risk for proarrhythmia secondary to the class II and class III antiarrhythmic properties (combined brady‐ and ventricular‐proarrhythmia) has also been shown in the meta‐analysis by Valembois et al.^8^ to be increased for dronedarone (RR = 1.95 [95% confidence interval: 0.77, 4.98]) and sotalol (3.55 [2.16, 5.83]) with placebo as a common comparator. The meta‐analysis by Freemantle et al.^7^ showed similar results (odds ratio = 1.45 [95% confidence interval: 1.02, 2.08] for dronedarone; 6.44 [1.03, 40.24] for sotalol). The results from this NMA are consistent with those studies, albeit with a numerically lower point estimate in the all‐studies NMA compared with RCTs‐only data. This may point to a difference that exists in RWE versus evidence from RCTs. However, no test of significance was applied to compare findings between all‐studies and RCTs‐only analyses, and therefore further investigation is warranted to confirm this finding. Of note, the type of proarrhythmic events reported in Valembois et al.^8^ differed between treatments: sotalol (61% ventricular events, 39% bradycardia) and dronedarone (41% ventricular events, 59% bradycardia). In the present analysis, there was also evidence from four studies suggesting a 51% lower risk of conduction disorders in patients treated with dronedarone versus sotalol; although this finding failed to reach significance, perhaps due to insufficient statistical power. The meta‐analyses by Valembois et al.^8^ and Freemantle et al.^7^ did not report on hospitalization, whereas this NMA included data on hospitalization and myocardial infarction. The data are consistent with numerically lower rates for dronedarone versus sotalol for these outcomes as derived from observational studies, despite failing to reach statistical significance. These findings may warrant a need for further investigations using real‐world data.

While both ESC^3^ and AHA/ACC/HRS^4^ guidelines emphasize that safety should primarily guide the selection of AAD therapy in AF, the AIM‐AF survey of US and EU physicians found efficacy to be the most important consideration for AAD selection.^9^ In spite of a recent change in recommendation from class IA to IIbA for sotalol in the ESC AF guidelines due to its safety profile, the survey study noted significant use of sotalol in general but also in specific patient populations of concern, such as left ventricular hypertrophy, or HFrEF.^9^ ESC guidelines state that dronedarone has the most solid safety data, but is not indicated in patients with decompensated heart failure, HFrEF, or patients with permanent AF.^3^ The use of RWE provides a measure of external validity that helps to bridge the gap between clinical practice and the well‐powered RCTs that provide the evidence base for clinical treatment guidelines.^9^ Both approaches applied to well‐designed and well‐powered studies of safety and efficacy will ultimately benefit the judicious use of AAD therapy in AF.

Noteworthy, our review excluded studies of patients with permanent AF. In the PALLAS trial,^42^ dronedarone significantly increased rates of heart failure, stroke, and CV death compared with placebo in patients with permanent AF. A later subanalysis demonstrated that concomitant treatment with digoxin could likely explain the increase in CV death seen with dronedarone.^43^ These results are in stark contrast to the findings of the ATHENA trial, conducted in patients with nonpermanent AF.^41^ ATHENA reported a significant reduction in unplanned CV hospitalization or death and significant reductions in rates of CV death and stroke, without a significant increase in the rate of heart failure in patients with paroxysmal or persistent (nonpermanent) AF treated with dronedarone. Consistent with the indicated population in ATHENA, the present meta‐analysis supports the positive safety results of dronedarone in patients with nonpermanent AF, but on a larger scale of multiple published RCTs as well as RWE studies. Another consideration is that most of the studies included in our review enrolled patients with normal to moderate LVEF dysfunction, while severe LVEF dysfunction was very rare. This observation aligns with clinical practice. Dronedarone and sotalol are not recommended in AF guidelines,^3^, ^4^ and are contraindicated in AF patients with New York Heart Association (NYHA) class IV heart failure or severe LVEF dysfunction (in the absence of an implantable cardioverter defibrillator with sotalol).^44^, ^45^ However, physicians may consider dronedarone or sotalol in favor of class 1C AADs in the presence of mildly impaired but stable LVEF including HFpEF.^3^ In the case of moderate to severe LVEF dysfunction, amiodarone is recommended. Taken together, it is reasonable to conclude based on available data, that the presence of permanent AF and/or unstable heart failure (NYHA class IV) or severe LVEF dysfunction are reasonable contraindications for dronedarone; however, safety benefits are apparent in patients with nonpermanent AF.

This review has several strengths. To our knowledge, this is the first SLR to combine published RCT and nonrandomized clinical trial data, with RWE from observational studies to summarize the comparative safety and efficacy of dronedarone versus sotalol in AF. The literature search was comprehensive and included three major electronic databases as well as several gray literature sources. This evidence base is representative of the current published quantitative analyses on this topic. Potential limitations of this review included restriction of the inclusion criteria to articles published only in the English language, which in addition to language bias has the potential to introduce the risk of ignoring key data, as well as missing cultural contexts. We consider that this bias is likely minimal, as many studies included in this review included patients/subjects from jurisdictions outside of the US and EU. Other limitations related to the included studies included the tendency for the methods of ascertainment of AF recurrence to vary, and heterogeneity in the length of follow‐up. As well, most studies included in this review reported a treatment duration of only several months, or did not report treatment duration at all. Further prospective multicenter RCTs with sufficient follow‐up are needed to confirm these findings.

## 5 CONCLUSIONS

Dronedarone, compared with sotalol, was associated with significantly lowered risk of all‐cause death in the analysis combining RCTs, a nonrandomized clinical trial, and observational studies, with no differences in AF recurrence observed between the two therapies. This meta‐analysis provides a comprehensive assessment of safety and efficacy evidence useful in evaluating treatment options in AF.

## CONFLICTS OF INTEREST STATEMENT

Jagmeet P. Singh receives consultation fees from Abbott Inc, Biotronik Inc, Boston Scientific, Cardiologs Inc, CVRx Inc, Cardiac Rhythm Group, EBR Inc, Impulse Dynamics, Implicity Inc, Medtronic Inc, Medscape Inc, Microport Inc, New Century Health, Nopras Inc, Orchestra BioMed Inc, Octagos Health Inc, and Sanofi.

Carina Blomström‐Lundqvist has received grants or personal fees from Medtronic Inc, Boston Scientific, Sanofi, Bayer, MSD, Bristol Myers Squibb, Johnson & Johnson, Boehringer Ingelheim, Philips, CathPrint, Milestone, and Abbott.

Mintu P. Turakhia has received grants or personal fees from Medtronic Inc, Abbott, Bristol Myers Squibb, American Heart Association, Biotronik Inc, Pfizer, Apple, Bayer, Myokardia, Johnson & Johnson, Milestone Pharmaceuticals, InCarda Pharmaceuticals, 100Plus, AliveCor, Sanofi, Gilead Sciences, and the Food and Drug Administration, and he is an employee of iRhythm Technologies, Inc. outside of the submitted work.

A. John Camm has received grants or personal fees from Abbott, Medtronic Inc, Boston Scientific, Biotronik Inc, Sanofi, Johnson & Johnson, Incarda Pharmaceuticals, Acesion, Arca, Bayer, Boehringer Ingelheim, Daiichi Sankyo, Menarini, Pfizer, and Bristol Myers Squibb. Dr. Camm is the editor‐in‐chief of *Clinical Cardiology*; however, he has recused himself from the peer‐review, editorial, and selection process conducted by the journal.

Mir Sohail Fazeli and Christopher Crotty are, or were, employed by Evidinno Outcomes Research Inc. (Vancouver, BC, Canada) at the time of this study, which was contracted by Sanofi to conduct this study.

Bahij Kreidieh reports no conflict of interest.

Peter R. Kowey receives consultation fees from Sanofi.

## Supporting information


**Supporting Information**.

## Data Availability

The data that support the findings of this study are available from the corresponding author upon reasonable request.
