# Supplementary material for: Dronedarone versus sotalol in patients with atrial fibrillation: A systematic literature review and network meta‐analysis
Source: Clin Cardiol. 2023 Apr 6;46(6):589–97. doi: 10.1002/clc.24011 (PMC10270269; doi:10.1002/clc.24011)
Supplement: Supplementary file 1 — Supporting Information. [file CLC-46-589-s001.docx]

**Supplementary Materials**

**Supplementary Table A.1**. Search strategy for MEDLINE® via OvidSP

| **Database: Ovid MEDLINE(R) ALL 1946 to June 15, 2021**  **Search executed: June 16, 2021** | | |
| --- | --- | --- |
| **#** | **String** | **Hits** |
| 1 | exp atrial fibrillation/ | 59439 |
| 2 | ((atrium or atrial or atria or auricular) adj2 (fibrillation or fibrilation)).ti,ab. | 76680 |
| 3 | or/1-2 | 88538 |
| 4 | exp dronedarone/ | 446 |
| 5 | (dronedarone or multaq or "2 butyl 3 [4 (3 dibutylaminopropoxy)benzoyl] 5 methanesulfonamidobenzofuran" or "n [2 butyl 3 [4 [3 (dibutylamino)propoxy]benzoyl] 5 benzofuranyl]methanesulfonamide" or "sr 33589" or "sr33589").ti,ab. | 629 |
| 6 | exp sotalol/ | 2075 |
| 7 | (sosotalol or alosot or "beta-cardone" or betacardone or betades or betapace or "bmy 5763 1" or "bmy 57631" or "bmy5763 1" or "bmy57631" or bonpro or corsotalol or darob or dexsotalol or dextrosotalol or "dl 4 (2 isopropylamino 1 hydroxyethyl) methanesulfonanilide hydrochloride" or favorex or gilucor or hipecor or isotalol or jutalex or levosotalol or "mj 1999" or "mj 5763 1" or rentibloc or rotalol or "so aqueous" or solavert or sorine or "sota saar" or sotab or sotabeta or sotaco? or sotahexal or sotalex or sotap?r or sotastad or sotylize or tachytalol or "2 isopropylamino 1 hydroxyethyl)methane sulfonanilide" or "(2 isopropylamino 1 hydroxyethyl)methane sulfonanilide hydrochloride" or "(2 isopropylamino 1 hydroxyethyl)methanesulfonanilide hydrochloride" or "4 (2 isopropyl 1 hydroxyethyl)methanesulfonanilide" or "4 (2 isopropylamine 1 hydroxyethyl)methanesulfonanilide" or "4 (2 isopropylamino 1 hydroxyethyl)methane sulfonanilide" or "4 (2 isopropylamino 1 hydroxyethyl)methanesulfoanilide" or "4 (2 isopropylamino 1 hydroxyethyl)methanesulfonamide" or "4 (2 isopropylamino 1 hydroxyethyl)methanesulfonanilide" or "4 (2 isopropylamino 1 hydroxyethyl)methanesulfonanilide hydrochloride" or "4 (2 isopropylamino) 1 hydroxyethylmethanesulfonilide hydrochloride" or "4 (isopropylamino 1 hydroxyethyl) methanesulfonanilide hydrochloride" or "4' (2 isopropylamine 1 hydroxyethyl)methane sulfonanilide" or "4' [1 hydroxy 2 (isopropylamine)ethyl]methanesulfonanilide hydrochloride" or "dl 4 (2 isopropylamino 1 hydroxyethyl) methanesulfonanilide hydrochloride" or "l 4 (2 isopropylamino 1 hydroxyethyl)methanesulfonamide" or "levo 4' [1 hydroxy 2 (isopropylamino)ethyl]methanesulfonanilide").ti,ab. | 2876 |
| 8 | or/4-7 | 3959 |
| 9 | 3 and 8 | 1057 |
| 10 | (exp animal/ or nonhuman/) not exp human/ | 4841883 |
| 11 | (editorial or letter or comment or news or newspaper article or interview or historical article).pt. | 2541576 |
| 12 | Case Study/ | 2185654 |
| 13 | case report.tw. | 336713 |
| 14 | or/10-13 | 9277951 |
| 15 | 9 not 14 | 821 |
| 16 | limit 15 to english language | 716 |

**Supplementary Table A.2**. Search strategy for Embase® via OvidSP

| **Database: Embase 1974 to 2021 June 15**  **Search executed: June 16, 2021** | | |
| --- | --- | --- |
| **#** | **String** | **Hits** |
| 1 | exp atrial fibrillation/ | 84793 |
| 2 | ((atrium or atrial or atria or auricular) adj2 (fibrillation or fibrilation)).ti,ab. | 136178 |
| 3 | or/1-2 | 157846 |
| 4 | exp dronedarone/ | 2334 |
| 5 | (dronedarone or multaq or "2 butyl 3 [4 (3 dibutylaminopropoxy)benzoyl] 5 methanesulfonamidobenzofuran" or "n [2 butyl 3 [4 [3 (dibutylamino)propoxy]benzoyl] 5 benzofuranyl]methanesulfonamide" or "sr 33589" or "sr33589").ti,ab. | 1030 |
| 6 | exp sotalol/ | 13325 |
| 7 | (sosotalol or alosot or "beta-cardone" or betacardone or betades or betapace or "bmy 5763 1" or "bmy 57631" or "bmy5763 1" or "bmy57631" or bonpro or corsotalol or darob or dexsotalol or dextrosotalol or "dl 4 (2 isopropylamino 1 hydroxyethyl) methanesulfonanilide hydrochloride" or favorex or gilucor or hipecor or isotalol or jutalex or levosotalol or "mj 1999" or "mj 5763 1" or rentibloc or rotalol or "so aqueous" or solavert or sorine or "sota saar" or sotab or sotabeta or sotaco? or sotahexal or sotalex or sotap?r or sotastad or sotylize or tachytalol or "2 isopropylamino 1 hydroxyethyl)methane sulfonanilide" or "(2 isopropylamino 1 hydroxyethyl)methane sulfonanilide hydrochloride" or "(2 isopropylamino 1 hydroxyethyl)methanesulfonanilide hydrochloride" or "4 (2 isopropyl 1 hydroxyethyl)methanesulfonanilide" or "4 (2 isopropylamine 1 hydroxyethyl)methanesulfonanilide" or "4 (2 isopropylamino 1 hydroxyethyl)methane sulfonanilide" or "4 (2 isopropylamino 1 hydroxyethyl)methanesulfoanilide" or "4 (2 isopropylamino 1 hydroxyethyl)methanesulfonamide" or "4 (2 isopropylamino 1 hydroxyethyl)methanesulfonanilide" or "4 (2 isopropylamino 1 hydroxyethyl)methanesulfonanilide hydrochloride" or "4 (2 isopropylamino) 1 hydroxyethylmethanesulfonilide hydrochloride" or "4 (isopropylamino 1 hydroxyethyl) methanesulfonanilide hydrochloride" or "4' (2 isopropylamine 1 hydroxyethyl)methane sulfonanilide" or "4' [1 hydroxy 2 (isopropylamine)ethyl]methanesulfonanilide hydrochloride" or "dl 4 (2 isopropylamino 1 hydroxyethyl) methanesulfonanilide hydrochloride" or "l 4 (2 isopropylamino 1 hydroxyethyl)methanesulfonamide" or "levo 4' [1 hydroxy 2 (isopropylamino)ethyl]methanesulfonanilide").ti,ab. | 3910 |
| 8 | or/4-7 | 15257 |
| 9 | 3 and 8 | 4191 |
| 10 | (conference or conference abstract or conference review).pt. | 4879773 |
| 11 | limit 10 to yr="2019-current" | 726951 |
| 12 | 10 not 11 | 4152822 |
| 13 | (exp animal/ or nonhuman/) not exp human/ | 6602396 |
| 14 | (book or chapter or editorial or erratum or letter or note or short survey or tombstone or comment).pt. | 3363539 |
| 15 | Case Study/ | 78908 |
| 16 | case report.tw. | 451500 |
| 17 | or/12-16 | 13871048 |
| 18 | 9 not 17 | 3132 |
| 19 | limit 18 to english language | 2856 |

**Supplementary Table A.3**. Search strategy for CENTRAL via OvidSP

| **Database: EBM Reviews - Cochrane Central Register of Controlled Trials May 2021**  **Search executed: June 16, 2021** | | |
| --- | --- | --- |
| **#** | **String** | **Hits** |
| 1 | exp atrial fibrillation/ | 4849 |
| 2 | ((atrium or atrial or atria or auricular) adj2 (fibrillation or fibrilation)).ti,ab. | 13029 |
| 3 | or/1-2 | 13400 |
| 4 | (dronedarone or multaq or "2 butyl 3 [4 (3 dibutylaminopropoxy)benzoyl] 5 methanesulfonamidobenzofuran" or "n [2 butyl 3 [4 [3 (dibutylamino)propoxy]benzoyl] 5 benzofuranyl]methanesulfonamide" or "sr 33589" or "sr33589").ti,ab. | 127 |
| 5 | exp sotalol/ | 303 |
| 6 | (s?talol or alosot or "beta-cardone" or betacardone or betades or betapace or "bmy 5763 1" or "bmy 57631" or "bmy5763 1" or "bmy57631" or bonpro or corsotalol or darob or dexsotalol or dextrosotalol or "dl 4 (2 isopropylamino 1 hydroxyethyl) methanesulfonanilide hydrochloride" or favorex or gilucor or hipecor or isotalol or jutalex or levosotalol or "mj 1999" or "mj 5763 1" or rentibloc or rotalol or "so aqueous" or solavert or sorine or "sota saar" or sotab or sotabeta or sotaco? or sotahexal or sotalex or sotap?r or sotastad or sotylize or tachytalol or "2 isopropylamino 1 hydroxyethyl)methane sulfonanilide" or "(2 isopropylamino 1 hydroxyethyl)methane sulfonanilide hydrochloride" or "(2 isopropylamino 1 hydroxyethyl)methanesulfonanilide hydrochloride" or "4 (2 isopropyl 1 hydroxyethyl)methanesulfonanilide" or "4 (2 isopropylamine 1 hydroxyethyl)methanesulfonanilide" or "4 (2 isopropylamino 1 hydroxyethyl)methane sulfonanilide" or "4 (2 isopropylamino 1 hydroxyethyl)methanesulfoanilide" or "4 (2 isopropylamino 1 hydroxyethyl)methanesulfonamide" or "4 (2 isopropylamino 1 hydroxyethyl)methanesulfonanilide" or "4 (2 isopropylamino 1 hydroxyethyl)methanesulfonanilide hydrochloride" or "4 (2 isopropylamino) 1 hydroxyethylmethanesulfonilide hydrochloride" or "4 (isopropylamino 1 hydroxyethyl) methanesulfonanilide hydrochloride" or "4' (2 isopropylamine 1 hydroxyethyl)methane sulfonanilide" or "4' [1 hydroxy 2 (isopropylamine)ethyl]methanesulfonanilide hydrochloride" or "dl 4 (2 isopropylamino 1 hydroxyethyl) methanesulfonanilide hydrochloride" or "l 4 (2 isopropylamino 1 hydroxyethyl)methanesulfonamide" or "levo 4' [1 hydroxy 2 (isopropylamino)ethyl]methanesulfonanilide").ti,ab. | 4410 |
| 7 | or/4-6 | 4575 |
| 8 | 3 and 7 | 323 |
| 9 | limit 8 to embase | 89 |
| 10 | limit 8 to medline | 190 |
| 11 | or/9-10 | 279 |
| 12 | 8 not 11 | 44 |
| 13 | limit 12 to english | 4 |

**Supplementary Table A.4**. Studies included in the review and screened for inclusion in the network meta-analysis

| **First Author & Year (Study Name)** | **Title** |
| --- | --- |
| **RCTs (Included in NMA)** | |
| [AFFIRM First Antiarrhythmic Drug Substudy Investigators](https://pubmed.ncbi.nlm.nih.gov/?sort=date&term=AFFIRM+First+Antiarrhythmic+Drug+Substudy+Investigators%5BCorporate+Author%5D) 2003 (AFFIRM) | Maintenance of sinus rhythm in patients with atrial fibrillation: an AFFIRM substudy of the first antiarrhythmic drug |
| Bellandi 2001 | Propafenone and sotalol in the prevention of paroxysmal atrial fibrillation: Long-term safety and efficacy study |
| Benditt 1999 | Maintenance of sinus rhythm with oral d,l-sotalol therapy in patients with symptomatic atrial fibrillation and/or atrial flutter |
| Capucci 2008 (DAPHNE) | The drug and pace health clinical evaluation (DAPHNE) study: A randomized trial comparing sotalol versus beta-blockers to treat symptomatic atrial fibrillation in patients with brady-tachycardia syndrome implanted with an antitachycardia pacemaker |
| Chun 2014 | Efficacy of dronedarone versus propafenone in the maintenance of sinus rhythm in patients with atrial fibrillation after electrical cardioversion |
| de Paola 1999 | Efficacy and safety of sotalol versus quinidine for the maintenance of sinus rhythm after conversion of atrial fibrillation |
| Fetsch 2004 (PAFAC) | Prevention of atrial fibrillation after cardioversion: Results of the PAFAC trial |
| Hohnloser 1995 | Efficacy and proarrhythmic hazards of pharmacologic cardioversion of atrial fibrillation: Prospective comparison of sotalol versus quinidine |
| Hohnloser 2009 (ATHENA) | Effect of dronedarone on cardiovascular events in atrial fibrillation |
| Juul-Moller 1990 | Sotalol versus quinidine for the maintenance of sinus rhythm after direct current conversion of atrial fibrillation |
| Kochiadakis 2000 | Low dose amiodarone and sotalol in the treatment of recurrent, symptomatic atrial fibrillation: A comparative, placebo controlled study |
| Le Heuzey 2010 (DIONYSOS) | A short-term, randomized, double-blind, parallel-group study to evaluate the efficacy and safety of dronedarone versus amiodarone in patients with persistent atrial fibrillation: The DIONYSOS study |
| Lee 1997 | Comparisons of oral propafenone and sotalol as an initial treatment in patients with symptomatic paroxysmal atrial fibrillation |
| Lombardi 2006 (A-COMET-II) | Azimilide vs. placebo and sotalol for persistent atrial fibrillation: The A-COMET-II (azimilide-cardioversion maintenance trial-II) trial |
| (ODYSSEUS) | Effects of dronedarone on cardiac geometry and function in patients with atrial fibrillation and left atrial enlargement (ODYSSEUS) |
| Patten 2004 (SOPAT) | Suppression of paroxysmal atrial tachyarrhythmias - results of the SOPAT trial |
| Plewan 2001 | Maintenance of sinus rhythm after electrical cardioversion of persistent atrial fibrillation: Sotalol vs bisoprolol |
| Reimold 1993 | Propafenone versus sotalol for suppression of recurrent symptomatic atrial fibrillation |
| Singh 1991 | Efficacy and safety of sotalol in digitalized patients with chronic atrial fibrillation |
| Singh 2005 (SAFE-T) | Amiodarone versus sotalol for atrial fibrillation |
| Singh 2007 (EURIDIS-ADONIS) | Dronedarone for maintenance of sinus rhythm in atrial fibrillation or flutter |
| Touboul 2003 (DAFNE) | Dronedarone for prevention of atrial fibrillation: A dose-ranging study |
| Vijayalakshmi 2006 | A randomized trial of prophylactic antiarrhythmic agents (amiodarone and sotalol) in patients with atrial fibrillation for whom direct current cardioversion is planned |
| **Non-randomized Clinical Trial (Included in NMA)** | |
| Antman 1990 | Therapy of refractory symptomatic atrial fibrillation and atrial flutter: A staged care approach with new antiarrhythmic drugs |
| **RCTs (Excluded from NMA)** | |
| Kochiadakis 2004 | Sotalol versus propafenone for long-term maintenance of normal sinus rhythm in patients with recurrent symptomatic atrial fibrillation |
| Reiffel 2015 (HARMONY) | The HARMONY trial: Combined ranolazine and dronedarone in the management of paroxysmal atrial fibrillation: Mechanistic and therapeutic synergism |
| (SOAR) | SOAR: Study observing antiarrhythmic remodelling using LGE-MRI (SOAR) |
| Wanless 1997 | Multicenter comparative study of the efficacy and safety of sotalol in the prophylactic treatment of patients with paroxysmal supraventricular tachyarrhythmias |
| **RWE (Included in NMA)** | |
| Agusala 2015 | Risk prediction for adverse events during initiation of sotalol and dofetilide for the treatment of atrial fibrillation |
| Andersen 2009 | Antiarrhythmic therapy and risk of death in patients with atrial fibrillation: A nationwide study |
| De Vecchis 2019 | High prevalence of proarrhythmic events in patients with history of atrial fibrillation undergoing a rhythm control strategy: A retrospective study |
| Friberg 2014 | Safety of dronedarone in routine clinical care |
| Friberg 2018 | Ventricular arrhythmia and death among atrial fibrillation patients using anti-arrhythmic drugs |
| Gao 2014 | Risk of cardiovascular events, stroke, congestive heart failure, interstitial lung disease, and acute liver injury: Dronedarone versus amiodarone and other antiarrhythmics |
| Gwag 2018 | Which antiarrhythmic drug to choose after electrical cardioversion: A study on non-valvular atrial fibrillation patients |
| Kim 2011 | One-year treatment persistence and potential adverse events among patients with atrial fibrillation treated with amiodarone or sotalol: A retrospective claims database analysis |
| Lee 2020 | Comparative clinical outcomes of dronedarone and sotalol in Asian patients with atrial fibrillation: A nationwide cohort study |
| Mascarenhas 2018 | Revisiting the role of antiarrhythmic drugs in prevention of atrial fibrillation recurrence: A single center retrospective review |
| Piccini 2014 | Comparison of safety of sotalol versus amiodarone in patients with atrial fibrillation and coronary artery disease |
| Sohns 2014 | Antiarrhythmic drug therapy for maintaining sinus rhythm early after pulmonary vein ablation in patients with symptomatic atrial fibrillation |
| Wharton 2020 | Comparative safety and effectiveness of sotalol versus dronedarone after catheter ablation for atrial fibrillation |
| **RWE (Excluded from NMA)** | |
| Aguilar-Shea 2016 | The safety and efficacy of sotalol in the management of acute atrial fibrillation: A retrospective case control study |
| Ehrlich 2019 | Impact of dronedarone on the risk of myocardial infarction and stroke in atrial fibrillation patients followed in general practices in Germany |
| Goehring 2020 | Outcomes associated with dronedarone use in patients with atrial fibrillation |
| Khachatryan 2021 | International cohort study on the effectiveness of dronedarone and other antiarrhythmic drugs for atrial fibrillation in real-world practice (EFFECT-AF) |
| La Pointe 2015 | Comparisons of hospitalization rates among younger atrial fibrillation patients receiving different antiarrhythmic drugs |
| Malladi 2021 | Association between specific antiarrhythmic drug prescription in the post-procedural blanking period and recurrent atrial arrhythmias after catheter ablation for atrial fibrillation |
| Noseworthy 2015 | Effect of antiarrhythmic drug initiation on readmission after catheter ablation for atrial fibrillation |
| Qin 2016 | Comparative effectiveness of antiarrhythmic drugs for rhythm control of atrial fibrillation |
| Taylor 2010 | Rhythm control agents and adverse events in patients with atrial fibrillation |

Abbreviations – NMA: Network meta-analysis; RCT: Randomized controlled trial; RWE: Real world evidence

**Supplementary Table A.5**. Studies excluded during full-text screening and reason for exclusion

| **First Author & Year** | **Title** | **Reason for Exclusion** |
| --- | --- | --- |
| Al-Jazairi 2021 | Antiarrhythmic drugs in patients with early persistent atrial fibrillation and heart failure: Results of the RACE 3 study | Study design |
| Amin 2012 | Temporal pattern and costs of rehospitalization in atrial fibrillation/atrial flutter patients with one or more additional risk factors | Intervention |
| Arya 2010 | First time and repeat cardioversion of atrial tachyarrhythmias - a comparison of outcomes | Outcomes |
| Bellandi 2002 | Comparing agents for prevention of atrial fibrillation recurrence | Other |
| Berg 2013 | Cost-effectiveness of dronedarone in patients with atrial fibrillation in the ATHENA trial | Study design |
| Blomstrom-Lundqvist 2020 | Efficacy and safety of dronedarone by atrial fibrillation history duration: Insights from the ATHENA study | Study design |
| Brignole 2003 | Rhythm versus rate control after ablation and pacing for paroxysmal atrial fibrillation: Clinical implications of the PAF 2 trial | Intervention |
| Campbell 2000 | Mortality in patients with atrial fibrillation – 1 year follow up of EMERALD (European and Australian multicenter evaluative research on atrial fibrillation dofetilide) | Intervention |
| Crijns 1991 | Serial antiarrhythmic drug treatment to maintain sinus rhythm after electrical cardioversion for chronic atrial fibrillation or atrial flutter | Intervention |
| Curtis 2020 | Efficacy and safety of dronedarone vs placebo across age and sex subgroups: A post hoc analysis of the ATHENA study among patients with nonpermanent atrial fibrillation/flutter | Study design |
| D'Angelo 2021 | Limited duration of antiarrhythmic drug use for newly diagnosed atrial fibrillation in a nationwide population under age 65 | Study design |
| Duytschaever 1998 | Factors influencing long term persistence of sinus rhythm after a first electrical cardioversion for atrial fibrillation | Outcomes |
| Ezekowitz 2015 | A placebo-controlled, double-blind, randomized, multicenter study to assess the effects of dronedarone 400 mg twice daily for 12 weeks on atrial fibrillation burden in subjects with permanent pacemakers | Population |
| Gao 2011 | Evaluation of dronedarone use in the us patient population between 2009 and 2010: A descriptive study using a claims database | Outcomes |
| Guerra 2014 | Efficacy and safety of dronedarone in patients previously treated with other antiarrhythmic agents | Study design |
| Halinen 1995 | Comparison of sotalol with digoxin-quinidine for conversion of acute atrial fibrillation to sinus rhythm (the Sotalol-Digoxin-Quinidine trial) | Outcomes |
| Hauser 2003 | Safety and feasibility of a clinical pathway for the outpatient initiation of antiarrhythmic medications in patients with atrial fibrillation or atrial flutter | Intervention |
| Heuzey 2010 | The RecordAF study: Design, baseline data, and profile of patients according to chosen treatment strategy for atrial fibrillation | Intervention |
| Ho 2005 | Effect of concomitant antiarrhythmic therapy on survival in patients with implantable cardioverter defibrillators | Population |
| Hohnloser 2010 | Dronedarone in patients with congestive heart failure: Insights from ATHENA | Study design |
| Johnson 2016 | Mortality risk of sotalol and amiodarone for post-CABG atrial fibrillation | Population |
| Joseph 2000 | A prospective, randomized controlled trial comparing the efficacy and safety of sotalol, amiodarone, and digoxin for the reversion of new-onset atrial fibrillation | Outcomes |
| Kabbani 2005 | Ablation of atrial fibrillation using microwave energy - early experience | Intervention |
| Kerr 1998 | The Canadian registry of atrial fibrillation: A noninterventional follow-up of patients after the first diagnosis of atrial fibrillation | Intervention |
| Khachatryan 2019 | International multicentre cohort study on relative effectiveness of dronedarone and other antiarrhythmic drugs for atrial fibrillation in real world practice (EFFECT-AF) | Duplicate publication |
| Kim 2014 | Impact of dronedarone treatment on healthcare resource utilization in patients with atrial fibrillation/flutter | Intervention |
| Malladi 2020 | Association between antiarrhythmic drug therapy prescription in the blanking period and recurrent atrial arrhythmias after first-time catheter ablation for atrial fibrillation | Duplicate publication |
| Opolski 2004 | Rate control vs rhythm control in patients with nonvalvular persistent atrial fibrillation: The results of the polish how to treat chronic atrial fibrillation (HOT CAFE) study | Intervention |
| Page 2011 | Rhythm- and rate-controlling effects of dronedarone in patients with atrial fibrillation (from the ATHENA trial) | Study design |
| Perrone 2018 | Efficacy and safety of dronedarone in patients with amiodarone-induced hyperthyroidism: A clinical study | Population |
| Pisters 2014 | Effect of dronedarone on clinical end points in patients with atrial fibrillation and coronary heart disease: Insights from the ATHENA trial | Study design |
| Reimold 1995 | Risk factors for the development of recurrent atrial fibrillation: Role of pacing and clinical variables | Intervention |
| Reynolds 2013 | Estimation of potential cost savings associated with reduced rates of cardiovascular hospitalization among atrial fibrillation/flutter patients treated with dronedarone in the ATHENA trial | Study design |
| Roy 2000 | Amiodarone to prevent recurrence of atrial fibrillation | Intervention |
| Saksena 2011 | Cardiovascular outcomes in the affirm trial (atrial fibrillation follow-up investigation of rhythm management): An assessment of individual antiarrhythmic drug therapies compared with rate control with propensity score-matched analyses | Intervention |
| Shantha 2019 | Antiarrhythmic drug therapy and all-cause mortality after catheter ablation for atrial fibrillation: A propensity matched analysis | Intervention |
| Steeds 1999 | An open label, randomised, crossover study comparing sotalol and atenolol in the treatment of symptomatic paroxysmal atrial fibrillation | Outcomes |
| Steinberg 2014 | Use of antiarrhythmic drug therapy and clinical outcomes in older patients with concomitant atrial fibrillation and coronary artery disease | Intervention |
| Thind 2020 | Dronedarone treatment following cardioversion in patients with atrial fibrillation/flutter: A post hoc analysis of the EURIDIS and ADONIS trials | Study design |
| Torp-Pedersen 2011 | Impact of dronedarone on hospitalization burden in patients with atrial fibrillation: Results from the ATHENA study | Study design |
| Vamos 2020 | Efficacy and safety of dronedarone in patients with a prior ablation for atrial fibrillation/flutter: Insights from the ATHENA study | Study design |
| Vamos 2019 | Impact of ablation status on the efficacy and safety of dronedarone in patients with atrial fibrillation/flutter: A post-hoc analysis of the ATHENA trial | Study design |
| Vincenzo De Paola 1999 | Efficacy and safety of sotalol versus quinidine for the maintenance of sinus rhythm after conversion of atrial fibrillation | Duplicate publication |
| Wharton 2020 | Cardiovascular hospitalization and repeat ablation rates following catheter ablation among atrial fibrillation patients treated with dronedarone vs. sotalol | Duplicate publication |
| Wong 2002 | Management and outcome of patients with atrial fibrillation during acute myocardial infarction: The GUSTO-III experience | Study design |
| Trial ID: 2009-018215-53 | The effect of the addition of dronedarone to, versus increase of, existing conventional rate control medication on ventricular rate during paroxysmal or persistent atrial fibrillation (AFRODITE study) | Intervention |

**Supplementary Table A.6**. Summary of study characteristics

| **First Author & Year (Study Name)** | **Study Design** | **Blinding** | **Region** | **Setting** | **N** | **Follow-up, Months** | **Analysis Method, Efficacy** | **Analysis Method, Safety** | **Arrhythmia Monitoring Method** |
| --- | --- | --- | --- | --- | --- | --- | --- | --- | --- |
| **RCTs (Included in NMA)** | | | | | | | | | |
| [AFFIRM First Antiarrhythmic Drug Substudy Investigators](https://pubmed.ncbi.nlm.nih.gov/?sort=date&term=AFFIRM+First+Antiarrhythmic+Drug+Substudy+Investigators%5BCorporate+Author%5D) 2003 (AFFIRM) | RCT | Open-label | North America | Multi-center | 661 | 3.84 ± 1.30 years | ITT | ITT | Follow-up visit only |
| Bellandi 2001 | RCT | Double-blind | Europe | Single-center | 300 | 12 months | ITT | ITT | Follow-up visit only |
| Benditt 1999 | RCT | Double-blind | North America | Multi-center | 253 | 12 months | mITT, ITT | NR | Tele-ECG |
| Capucci 2008 (DAPHNE) | RCT | Single-blind | Europe | Multi-center | 135 | 19 (mean) months | ITT | ITT | N/A |
| de Paola 1999 | RCT | Open-label | South America | Multi-center | 121 | 6 months | ITT | ITT | 24-hr Holter |
| Fetsch 2004 (PAFAC) | RCT | Double-blind | Europe | Multi-center | 848 | 2 years | ITT | ITT | 24-hr Holter; Tele-ECG |
| Hohnloser 1995 | RCT | Open-label | Europe | Single-center | 50 | 6 months | ITT | ITT | Follow-up visit only |
| Hohnloser 2009 (ATHENA) | RCT | Double-blind | Various | Multi-center | 4628 | 1 – 2.5 years | ITT | ITT | NR |
| Juul-Moller 1990 | RCT | Open-label | Europe | Multi-center | 183 | 6 months | ITT | ITT | Follow-up visit only |
| Kochiadakis 2000 | RCT | Single-blind | Europe | Single-center | 186 | Amiodarone: 21.6 (mean); Sotalol: 22 (mean); Placebo: 22.3 (mean) months | NR | NR | Mixed* |
| Kwang Jin 2014 | RCT | Open-label | Asia | Single-center | 98 | 6 months | ITT | NR | Follow-up visit only |
| Le Heuzey 2010 (DIONYSOS) | RCT | Double-blind | Various | Multi-center | 504 | 190 days | ITT | ITT | Follow-up visit only |
| Lee 1997 | RCT | Double-blind | Asia | Single-center | 79 | ≥ 3 months | NR | NR | Other ambulatory device |
| Lombardi 2006 (A-COMET-II) | RCT | Double-blind | Various | Multi-center | 658 | 26 weeks | ITT | ITT | NR |
| NCT01198873 (ODYSSEUS) | RCT | Double-blind | North America | Multi-center | 76 | 12 months | mITT | ITT | NR |
| Patten 2004 (SOPAT) | RCT | Double-blind | Europe | Multi-center | 1033 | 12 months | ITT, PP | NR | Tele-ECG |
| Plewan 2001 | RCT | Open-label | Europe | NR | 128 | 12 months | ITT | NR | Follow-up visit only |
| Reimold 1993 | RCT | Open-label | North America | Single-center | 100 | 12 months | NR | NR | N/A |
| Singh 1991 | RCT | Double-blind | North America | Multi-center | 34 | 6 months | NR | NR | NR |
| Singh 2005 (SAFE-T) | RCT | Double-blind | North America | Multi-center | 665 | 12 – 54 months | ITT | ITT | Tele-ECG |
| Singh 2007 (EURIDIS-ADONIS) | RCT | Double-blind | Various | Multi-center | 1237 | 12 months | mITT | NR | Tele-ECG |
| Touboul 2003 (DAFNE) | RCT | Double-blind | NR | Multi-center | 199 | 6 months | ITT | NR | Tele-ECG |
| Vijayalakshmi 2006 | RCT | Open-label | Europe | Single-center | 94 | 6 months | ITT | ITT | NR |
| **Non-randomized Clinical Trial (Included in NMA)** | | | | | | | | | |
| Antman 1990 | Non-randomized clinical trial | Open-label | North America | Multi-center | 109 | Propafenone: 5.6 (mean); Sotalol: 3.9 (mean) months | NR | NR | Tele-ECG |
| **RCTs (Excluded from NMA)** | | | | | | | | | |
| Kochiadakis 2004 | RCT | Single-blind | Europe | Single-center | 254 | Sotalol: 25 ± 6, Propafenone: 25 ± 7 and Control group: 3 5± 6 months | NR | NR | Tele-ECG |
| Reiffel 2015 (HARMONY) | RCT | Double-blind | Various | Multi-center | 134 | 12 weeks | mITT | ITT | Implant |
| NCT01182376 (SOAR) | RCT | Double-blind | North America | NR | 33 | 12 months | NR | ITT | NR |
| Wanless 1997 | RCT | Double-blind | Various | Multi-center | 126 | 2, 4, or 8 months | mITT | NR | Tele-ECG |
| **RWE (Included in NMA)** | | | | | | | | | |
| Agusala 2015 | Retrospective cohort | NR | North America | Single-center | 329 | NR | NR | NR | NR |
| Andersen 2009 | Retrospective cohort | NR | Europe | Multi-center | 141500 | 3.2 (mean) years | NR | NR | N/A |
| De Vecchis 2019 | Retrospective cohort | NR | Europe | Multi-center | 624 | 20 months | NR | NR | NR |
| Friberg 2014 | Retrospective cohort | NR | Europe | Multi-center | 174995 | 1.6 (mean) years | ITT | ITT | N/A |
| Friberg 2018 | Retrospective cohort | NR | Europe | Multi-center | 312341 | 1 (minimum) years | NR | NR | NR |
| Gao 2014 | Retrospective cohort | NR | North America | Multi-center | 8,523 | < 1.5 years | NR | NR | N/A |
| Gwag 2018 | Retrospective cohort | NR | Asia | Single-center | 286 | 1 years | NR | NR | Mixed* |
| Kim 2011 | Retrospective cohort | NR | North America | Multi-center | 3459 | 12 months | NR | NR | NR |
| Lee 2020 | Retrospective cohort | NR | Asia | Multi-center | 4694 | 335 (mean) days; Dronedarone 368; Sotalol 270 days | NR | NR | NR |
| Mascarenhas 2018 | Retrospective cohort | NR | North America | Single-center | 145 | NR | NR | NR | Implant |
| Piccini 2014 | Retrospective cohort | NR | North America | Single-center | 2838 | 4.2 (mean) years; Sotalol 7.5 (median); Amiodarone 4.0 (median); no AAD 4.1 (median) years | NR | NR | N/A |
| Sohns 2014 | Retrospective cohort | NR | Europe | Single-center | 274 | 2 months | NR | NR | Tele-ECG |
| Wharton 2020 | Retrospective cohort | NR | North America | Multi-center | 3630 | 6 months | NR | NR | NR |
| **RWE (Excluded from NMA)** | | | | | | | | | |
| Aguilar-Shea 2016 | Case-control | NR | Europe | Single-center | 300 | 2 years | NR | NR | N/A |
| Ehrlich 2019 | Retrospective cohort | NR | Europe | Multi-center | 21222 | 6 years | NR | NR | N/A |
| Goehring 2020 | Retrospective cohort | NR | North America | Multi-center | 32571 | 6.6 months | NR | NR | Follow-up visit only |
| Khatchatryan 2021 (EFFECT AF) | Prospective cohort | NR | Various | Multi-center | 1009 | 12 – 18 months | NR | NR | NR |
| La Pointe 2015 | Retrospective cohort | NR | North America | Multi-center | 8562 | NR | NR | NR | N/A |
| Malladi 2021 | Retrospective cohort | NR | North America | Single-center | 478 | 37.5 months | NR | NR | Mixed* |
| Noseworthy 2015 | Retrospective cohort | NR | North America | Multi-center | 2542 | NR | NR | NR | N/A |
| Qin 2016 | Retrospective cohort | NR | North America | Single-center | 5952 | 26.1 (mean) months | NR | NR | Mixed* |
| Taylor 2010 | Case-control | NR | Europe | Multi-center | 14929 | NR | NR | NR | N/A |

Abbreviations – ECG: Electrocardiogram; ITT: Intention to treat; mITT: Modified intention to treat; N/A: Not applicable; NMA: Network meta-analysis; NR: Not reported; PP: Per protocol; RCT Randomized controlled trial; RWE: Real world evidence.

*Studies that used a mixture of ambulatory (e.g., 24-hr ambulatory monitor or implantable device) and in-clinic methods (e.g., in-clinic electrocardiogram) to measure arrythmia-related end points.

**Supplementary Table A.7**. Summary of intervention characteristics

| **First Author & Year (Study Name)** | **Treatment** | **N** | **Dose** | **Frequency of Administration** | **Duration of Treatment** | **Treatment Discontinuation** |
| --- | --- | --- | --- | --- | --- | --- |
| **RCTs (Included in NMA)** | | | | | | |
| [AFFIRM First Antiarrhythmic Drug Substudy Investigators](https://pubmed.ncbi.nlm.nih.gov/?sort=date&term=AFFIRM+First+Antiarrhythmic+Drug+Substudy+Investigators%5BCorporate+Author%5D) 2003 (AFFIRM) | Amiodarone | 106 | 200 mg | QD | NR | NR |
|  | Sotalol | 125 | 240 mg | QD | NR | NR |
| Bellandi 2001 | Placebo | 92 | NR | TID | NR | NR |
|  | Propafenone | 102 | 13 mg/kg/day | TID | NR | NR |
|  | Sotalol | 106 | 3 mg/kg/day | TID | NR | NR |
| Benditt 1999 | Placebo | 69 | NR | BID | 28 days | n = 53 [AE (n = 4), Adverse arrhythmic event (n = 3), other AE-Fatigue, dizziness, dyspnea, or weakness (n = 1), lack of efficacy-relapse (n = 46), withdrawal of consent (n = 2), other (n = 1)] |
|  | Sotalol | 59 | 80 mg | BID | 84 days | N = 46 [AE (n = 7), Adverse arrhythmic event (n = 1), bradycardia (n = 1), other AE-Fatigue, dizziness, dyspnea, or weakness (n = 5), lack of efficacy-relapse (n = 34), withdrawal of consent (n = 1), other (n = 4)] |
|  | Sotalol | 63 | 120 mg | BID | 80 days | n = 45 [AE (n = 11), Adverse arrhythmic event (n = 1), bradycardia (n = 1), QT prolonged (n = 3), intracerebral hemorrhage (n = 1), other AE-Fatigue, dizziness, dyspnea, or weakness (n = 5), lack of efficacy-relapse (n = 31), protocol deviation (n = 1), other (n = 2)] |
|  | Sotalol | 62 | 160 mg | BID | 32.5 days | n = 48 [AE (n = 18), Adverse arrhythmic event (n = 2), congestive heart failure (n = 2), bradycardia (n = 5), QT prolonged (n = 2), other AE-Fatigue, dizziness, dyspnea, or weakness (n = 7), lack of efficacy-relapse (n = 26), protocol deviation (n = 1), withdrawal of consent (n = 3)] |
| Capucci 2008 (DAPHNE) | Atenolol | 66 | 62 mg | QD | 12 months | NR |
|  | Metoprolol | 66 | 104 mg | QD | 12 months | NR |
|  | Sotalol | 69 | 167 mg/day | NR | 12 months | NR |
| de Paola 1999 | Quinidine | 63 | 200 mg | QID | 6 months | n = 10 (16%) Withdrawal (side effects) |
|  | Sotalol | 58 | 160 mg | BID | 6 months | n = 7 (12%) Withdrawal (side effects) |
| Fetsch 2004 (PAFAC) | Placebo | 88 | NR | NR | 12 months | NR |
|  | Sotalol | 383 | 80-160 mg | TID (80 mg), BID (160 mg) | 12 months | NR |
| Hohnloser 1995 | Quinidine | 25 | 700 mg | BID | 6 months | NR |
|  | Sotalol | 25 | 80 mg | BID | 6 months | NR |
| Hohnloser 2009 (ATHENA) | Dronedarone | 2301 | 400 mg | BID | 2 years | 12.7% AE, 7.5% subject's request, 9.4% other reasons |
|  | Placebo | 2327 | NR | NR | 2 years | 8.1% AE, 7.5% subject's request, 14.4% other reasons |
| Juul-Moller 1990 | Quinidine | 85 | 600 mg | BID | 6 months | 27% in total population, 26% in ITT population |
|  | Sotalol | 98 | 80 mg | BID | 6 months | 11% in total population, 11% in ITT population |
| Kochiadakis 2000 | Amiodarone | 65 | 200 mg | QD | 2 years | n = 11 AE |
|  | Placebo | 60 | NR | NR | 2 years | NR |
|  | Sotalol | 61 | 160–480 mg | QD | 2 years | n = 2 AE |
| Kwang Jin 2014 | Dronedarone | 49 | 400 mg | BID | NR | NR |
|  | Propafenone | 49 | 150 mg | TID | NR | NR |
| Le Heuzey 2010 (DIONYSOS) | Amiodarone | 255 | 600 mg | QD | 7 (median) months | n = 34 [Lack of efficacy (n = 0), intolerance (n = 34)] |
|  | Dronedarone | 249 | 400 mg | BID | 7 (median) months | n = 26 [Lack of efficacy (n = 1), intolerance (n = 25)] |
| Lee 1997 | Propafenone | 41 | 150 mg | every 6 hours | NR | n = 2 (5%) Intolerable side effects |
|  | Sotalol | 38 | 80 mg | every 12 hours | NR | n = 4 (11%) Intolerable side effects |
| Lombardi 2006 (A-COMET-II) | Azimilide | 211 | 125 mg | QD | 26 weeks | n = 9 Withdrawn from the study prior to start of the efficacy period for different reasons |
|  | Placebo | 224 | NR | NR | 26 weeks | n = 5 Withdrawn from the study prior to start of the efficacy period for different reasons |
|  | Sotalol | 223 | 160 mg | BID | 26 weeks | n = 7 Withdrawn from the study prior to start of the efficacy period for different reasons |
| NCT01198873 (ODYSSEUS) | Dronedarone | 35 | 400 mg | BID | NR | NR |
|  | Placebo | 41 | NR | NR | NR | NR |
| Patten 2004 (SOPAT) | Placebo | 251 | NR | NR | NR | NR |
|  | Sotalol | 264 | 320 mg | QD | 233 days | NR |
| Plewan 2001 | Bisoprolol | 64 | 5 mg | QD | NR | NR |
|  | Sotalol | 64 | 80 mg | BID | NR | NR |
| Reimold 1993 | Sotalol | 50 | 225 mg | TID | 12 | n = 4 Side effects |
|  | Propafenone | 50 | 160 mg | BID | 12 | n = 6 Side effects |
| Singh 1991 | Placebo | 10 | NR | QD | 6 months | NR |
|  | Sotalol | 24 | 80-320 mg | QD | 6 months | n = 3 [Asymptomatic bradycardia (n = 2), nausea and vomiting (n = 1)] |
| Singh 2005 (SAFE-T) | Amiodarone | 267 | 200 mg | QD | 4.5 years | n = 31 Withdrew consent, n = 11 lost to follow-up |
|  | Placebo | 137 | NR | NR | 4.5 years | n = 23 Withdrew consent, n = 5 lost to follow-up |
|  | Sotalol | 261 | 160 mg | BID | 4.5 years | n = 27 Withdrew consent, n = 12 lost to follow-up |
| Singh 2007 (EURIDIS) | Dronedarone | 411 | 400 mg | BID | 12 months | n = 67 NFI |
|  | Placebo | 201 | NR | BID |  | n = 25 NFI |
| Singh 2007 (ADONIS) | Dronedarone | 417 | 400 mg | BID | 12 months | n = 81 NFI |
|  | Placebo | 208 | NR | BID |  | n = 36 NFI |
| Touboul 2003 (DAFNE) | Dronedarone | 54 | 800 mg | BID | 6 months | n = 3 NFI |
|  | Dronedarone | 54 | 1200 mg | BID | 6 months | n = 5 NFI |
|  | Dronedarone | 43 | 1600 mg | BID | 6 months | n = 14 NFI |
|  | Placebo | 48 | NR | BID | 6 months | n = 0 NFI |
| Vijayalakshmi 2006 | Amiodarone | 27 | 200 mg | QD | 6 months | n = 1 NFI |
|  | Sotalol | 36 | 160 mg | BID | 6 months | n = 4 NFI |
| **Non-randomized Clinical Trial (Included in NMA)** | | | | | | |
| Antman 1990 | Propafenone | 109 | NR | NR | 5.6 months | NR |
|  | Sotalol | 48 | NR | NR | 3.9 months | NR |
| **RCTs (Excluded from NMA)** | | | | | | |
| Kochiadakis 2004 | Placebo | 83 | NR | TID | 30 months | NR |
|  | Propafenone | 86 | 150 mg | TID | 30 months | n = 5 Side effects |
|  | Sotalol | 85 | 80 mg | BID | 30 months | n = 5 Side effects |
| Reiffel 2015 (HARMONY) | Dronedarone | 26 | 225 mg | BID | 12 weeks | NR |
|  | Placebo | 26 | NR | BID | 12 weeks | NR |
|  | Ranolazine | 26 | 750 mg | BID | 12 weeks | NR |
|  | Ranolazine + Dronedarone | 26 | 750 mg + 150 mg | BID | 12 weeks | NR |
|  | Ranolazine + Dronedarone | 27 | 750 mg + 225 mg | BID | 12 weeks | NR |
| NCT01182376 (SOAR) | Dronedarone | 18 | NR | NR | NR | NR |
|  | Placebo | 15 | NR | NR | NR | NR |
| Wanless 1997 | Placebo | 35 | NR | BID | 2, 4, or 8 | n = 1 Syncopal episodes with rapid pulse: |
|  | Sotalol | 45 | 80 mg | BID | 2, 4, or 8 | n = 4 Typical B-blocking side effects, including bradycardia, dyspnea, and fatigue |
|  | Sotalol | 46 | 160 mg | BID | 2, 4, or 8 | n = 2 Typical B-blocking side effects, including bradycardia, dyspnea, and fatigue |
| **RWE (Included in NMA)** | | | | | | |
| Agusala 2015 | Dofetilide | 102 | NR | NR | NR | NR |
|  | Sotalol | 227 | 80 mg | BID | NR | NR |
| Andersen 2009 | Amiodarone | 10376 | 287.4 mg | NR | 1.6 years | NR |
|  | Sotalol | 3356 | 205.6 mg | NR | 2.4 years | NR |
|  | Propafenone | 3745 | 411.4 mg | NR | 2.3 years | NR |
|  | Flecainide | 23346 | 122.9 mg | NR | 1.7 years | NR |
| De Vecchis 2019 | Amiodarone | 230 | 200 mg | QD | 20 months | NR |
|  | Sotalol | 140 | 80 mg | BID | 20 months | NR |
| Friberg 2014 | Dronedarone | 4856 | NR | NR | 6 months | NR |
|  | Sotalol | 5722 | NR | NR | NR | NR |
|  | Amiodarone | 2809 | NR | NR | NR | NR |
|  | Propafenone | 57 | NR | NR | NR | NR |
|  | Flecainide | 2331 | NR | NR | NR | NR |
| Friberg 2018 | Dronedarone | 8254 | NR | NR | 1.4 years | NR |
|  | Amiodarone | 10541 | NR | NR | 1.5 years | NR |
|  | Sotalol | 16137 | NR | NR | 3.3 years | NR |
| Gao 2014 | Amiodarone | 2782 | NR | NR | 3.6 months | NR |
|  | Dronedarone | 1727 | NR | NR | 4 months | NR |
|  | Flecainide | 1502 | NR | NR | 4.5 months | NR |
|  | Propafenone | 1146 | NR | NR | 4.2 months | NR |
|  | Sotalol | 1366 | NR | NR | 5.3 months | NR |
| Gwag 2018 | Amiodarone | 128 | 200 mg | BID | 1 year | NR |
|  | Dronedarone | 40 | 400 mg | BID | 1 year | NR |
|  | Flecainide | 33 | 50 mg | BID | 1 year | NR |
|  | Propafenone | 64 | 300 mg | BID | 1 year | NR |
| Kim 2011 | Amiodarone | 2392 | NR | NR | NR | 30.6% of patients remained on index AF therapy after 360 days of follow-up |
|  | Sotalol | 1067 | NR | NR | NR | 53.2% of patients remained on index AF therapy after 360 days of follow-up |
| Lee 2020 | Dronedarone | 3119 | NR | NR | 368 days | n = 715 (29.0%) Failed to renew the index AAD within a defined grace period of 30-days |
|  | Sotalol | 1575 | NR | NR | 270 days | n = 358 (25.8%) Failed to renew the index AAD within a defined grace period of 30-days: |
| Mascarenhas 2018 | Amiodarone (AF recurrence) | 13 | 151.65 mg/day | NR | NR | NR |
|  | Amiodarone (no AF recurrence) | 52 | 217.34 mg/day | NR | NR | NR |
|  | Flecainide | 2 | NR | NR | NR | NR |
|  | Propafenone (AF recurrence) | 3 | 716.67 mg/day | NR | NR | NR |
|  | Propafenone (no AF recurrence) | 9 | 805.56 mg/day | NR | NR | NR |
|  | Sotalol (AF recurrence) | 17 | 146.38 mg/day | NR | NR | NR |
|  | Sotalol (no AF recurrence) | 49 | 209.18 mg/day | NR | NR | NR |
| Piccini 2014 | Amiodarone | 856 | NR | NR | NR | NR |
|  | Sotalol | 226 | NR | NR | NR | NR |
| Sohns 2014 | Amiodarone | 115 | 600 mg | QD | NR | NR |
|  | Bisoprolol | NR (n = 89 BBs) | 2.5-10 mg | QD | NR | NR |
|  | Dronedarone | 29 | 400 mg | BID | NR | NR |
|  | Flecainide | 99 | 100-200 mg | QD | NR | NR |
|  | Metoprolol | NR (n = 89 BBs) | 100-200 mg | QD | NR | NR |
|  | Sotalol | 37 | 80-160 mg | BID | NR | NR |
| Wharton 2020 | Dronedarone | 1815 | NR | NR | 1 | NR |
|  | Sotalol | 1815 | NR | NR | 1 | NR |
| **RWE (Excluded from NMA)** | | | | | | |
| Aguilar-Shea 2016 | Amiodarone | 66 | NR | NR | 2 years | NR |
|  | No treatment | 36 | NR | NR | 2 years | NR |
|  | Flecainide | 24 | NR | NR | 2 years | NR |
|  | Propafenone | 24 | NR | NR | 2 years | NR |
|  | Disopyramide | 24 | NR | NR | 2 years | NR |
|  | Digoxin | 75 | NR | NR | 2 years | NR |
|  | Beta-blocker | 75 | NR | NR | 2 years | NR |
|  | Verapamil | 75 | NR | NR | 2 years | NR |
|  | Diltiazem | 75 | NR | NR | 2 years | NR |
|  | Sotalol | 120 | 40-160 mg | BID | 2 years | NR |
| Ehrlich 2019 | Dronedarone | 3498 | NR | NR | 24.6 months | NR |
|  | Other AAD | 17724 | NR | NR | 23.5 months | NR |
| Goehring 2020 | Dronedarone | 6349 | NR | NR | 6.6 months | NR |
|  | Other AAD | 12698 | NR | NR | 6.5 months | NR |
| Khachatryan 2021 (EFFECT AF) | Dronedarone | 505 | NR | NR | NR | NR |
|  | Flecainide or Propafenone | 210 | NR | NR | NR | NR |
|  | Sotalol | 54 | NR | NR | NR | NR |
|  | Amiodarone | 232 | NR | NR | NR | NR |
|  | Ajmaline and quinidine | 3 | NR | NR | NR | NR |
| Malladi 2021 | Amiodarone | 65 | NR | NR | 126 days | NR |
|  | Dronedarone | 51 | NR | NR | 107 days | NR |
|  | No AAD | 71 | NR | NR | NR | NR |
|  | Propafenone or Flecainide | 126 | NR | NR | 90 days | NR |
|  | Sotalol or Dofetilide | 165 | NR | NR | 108 days | NR |
| Noseworthy 2015 | AAD | 519 | NR | NR | NR | NR |
|  | Amiodarone | 150 | NR | NR | NR | NR |
|  | Dronedarone | 102 | NR | NR | NR | NR |
|  | No AAD | 2023 | NR | NR | NR | NR |
|  | Propafenone or Flecainide | 145 | NR | NR | NR | NR |
|  | Sotalol or Dofetilide | 120 | NR | NR | NR | NR |
| Qin 2016 | Amiodarone | 2266 | NR | NR | NR | NR |
|  | Class 1C drug | 941 | NR | NR | NR | NR |
|  | Dofetilide | 539 | NR | NR | NR | NR |
|  | Dronedarone | 488 | NR | NR | NR | NR |
|  | Sotalol | 1718 | NR | NR | NR | NR |
| Taylor 2010 | Amiodarone | 8229 | NR | NR | NR | NR |
|  | Flecainide | 1335 | NR | NR | NR | NR |
|  | Sotalol | 5365 | NR | NR | NR | NR |

Abbreviations – AAD: Antiarrhythmic drug; AE: Adverse event; BID: Twice daily; ITT: Intention to treat; kg: Kilogram; mg: Milligram; NFI: No further information; NMA: Network meta-analysis; NR: Not reported; QD: Four times a day; RCT: Randomized controlled trial; RWE: Real world evidence; TID: Three times a day.

**Supplementary Table A.8**. Summary of patient baseline characteristics

| **First Author & Year (Study Name)** | **Age, Mean or Median, Years** | **Male, %** | **HT, %** | **Diabetes, %** | **Paroxysmal AF, %** | **Persistent AF, %** | **Valvular Heart Disease, %** | **CAD, %** | **COPD, %** | **Prior MI, %** | **LVEF, Mean or Median, %** |
| --- | --- | --- | --- | --- | --- | --- | --- | --- | --- | --- | --- |
| **RCTs (Included in NMA)** | | | | | | | | | | | |
| [AFFIRM First Antiarrhythmic Drug Substudy Investigators](https://pubmed.ncbi.nlm.nih.gov/?sort=date&term=AFFIRM+First+Antiarrhythmic+Drug+Substudy+Investigators%5BCorporate+Author%5D) 2003 (AFFIRM) | 70 | 63 | 51 | NR | NR | NR | NR | 15 | NR | 10 | NR |
| Bellandi 2001 | 52 | NR | 37 | NR | NR | NR | 15 | 20 | NR | NR | 55 |
| Benditt 1999 | 62 | 64 | NR | NR | 48 | NR | NR | NR | NR | 11 | NR |
| Capucci 2008 (DAPHNE) | 73 | 50 | 53 | NR | NR | NR | 10 | NR | NR | 7 | 55 |
| de Paola 1999 | 54 | 59 | 49 | NR | NR | NR | NR | NR | NR | NR | 68 |
| Fetsch 2004 (PAFAC) | 62 | 67 | NR | NR | NR | NR | NR | NR | NR | 5 | NR |
| Hohnloser 1995 | 63 | 35 | 20 | NR | NR | NR | 28 | 30 | NR | NR | NR |
| Hohnloser 2009 (ATHENA) | 72 | 53 | 86 | NR | NR | NR | 16 | NR | NR | NR | NR |
| Juul-Moller 1990 | 59 | 82 | 26 | NR | NR | NR | 5 | NR | NR | NR | NR |
| Kochiadakis 2000 | 63 | 52 | NR | NR | 40 | NR | NR | NR | NR | NR | 54 |
| Kwang Jin 2014 | 60 | 81 | 48 | 13 | NR | NR | NR | NR | NR | NR | 59 |
| Le Heuzey 2010 (DIONYSOS) | 64 | 71 | 67 | NR | 4 | 63 | 19 | NR | NR | NR | NR |
| Lee 1997 | 62 | 70 | 33 | NR | NR | NR | NR | 20 | NR | NR | NR |
| Lombardi 2006 (A-COMET-II) | 62 | 62 | 67 | NR | NR | NR | NR | NR | NR | NR | NR |
| NCT01198873 (ODYSSEUS) | 72 | 63 | NR | NR | NR | NR | NR | NR | NR | NR | NR |
| Patten 2004 (SOPAT) | 60 | 64 | 48 | NR | NR | NR | 13 | 16 | NR | 3 | 62 |
| Plewan 2001 | 59 | 63 | 31 | NR | NR | NR | 6 | 35 | NR | NR | 42 |
| Reimold 1993 | 62 | 61 | 19 | NR | 24 | NR | 30 | 16 | NR | NR | 60 |
| Singh 1991 | 60 | 70 | NR | NR | NR | NR | NR | NR | NR | NR | NR |
| Singh 2005 (SAFE-T) | 67 | 99 | 66 | 25 | NR | NR | 7 | NR | 12 | NR | 51 |
| Singh 2007 (EURIDIS-ADONIS) | 63 | 69 | 59 | NR | NR | NR | 11 | 20 | NR | NR | 60 |
| Touboul 2003 (DAFNE) | 63 | 68 | 52 | NR | NR | NR | 39 | 22 | NR | NR | 55 |
| Vijayalakshmi 2006 | 65 | 71 | 35 | 9 | NR | NR | NR | NR | NR | 6 | 43 |
| **Non-randomized Clinical Trial (Included in NMA)** | | | | | | | | | | | |
| Antman 1990 | 63 | NR | 36 | NR | 56 | NR | 29 | NR | NR | NR | 57 |
| **RCTs (Excluded from NMA)** | | | | | | | | | | | |
| Kochiadakis 2004 | 63 | 51 | NR | NR | 50 | NR | NR | NR | NR | NR | 53 |
| Reiffel 2015 (HARMONY) | 72 | 48 | 85 | 24 | NR | NR | NR | 33 | NR | NR | 57 |
| NCT01182376 (SOAR) | 67 | 64 | NR | NR | NR | NR | NR | NR | NR | NR | NR |
| Wanless 1997 | NR | NR | NR | NR | NR | NR | NR | NR | NR | NR | NR |
| **RWE (Included in NMA)** | | | | | | | | | | | |
| Agusala 2015 | 63 | 70 | 52 | 12 | 0 | 0 | 0 | 23 | NR | NR | 50 |
| Andersen 2009 | 66 | 61 | NR | 5 | NR | NR | NR | NR | 4 | 6 | NR |
| De Vecchis 2019 | NR | NR | NR | NR | NR | NR | NR | NR | NR | NR | NR |
| Friberg 2014 | 76 | 56 | 64 | 18 | NR | NR | 11 | NR | 8 | 18 | NR |
| Friberg 2018 | 70 | 60 | 57 | 16 | NR | NR | NR | NR | 5 | 16 | NR |
| Gao 2014 | 62 | 70 | 71 | 25 | NR | NR | NR | NR | NR | NR | NR |
| Gwag 2018 | 58 | 86 | 46 | 15 | 1 | 99 | NR | 8 | 2 |  | 59 |
| Kim 2011 | 71 | 62 | 47 | 22 | NR | NR | 27 | NR | NR | NR | NR |
| Lee 2020 | 63 | 66 | NR | NR | NR | NR | NR | NR | NR | NR | NR |
| Mascarenhas 2018 | 76 | 49 | 96 | 18 | 55 | 5 | 17 | 61 | NR | NR | 57 |
| Piccini 2014 | 68 | 69 | 71 | 31 | NR | NR | NR | NR | 9 | 48 | 54 |
| Sohns 2014 | 63 | 66 | NR | 7 | 67 | NR | NR | NR | NR | NR | 55 |
| Wharton 2020 | NR | NR | NR | NR | NR | NR | NR | NR | NR | NR | NR |
| **RWE (Excluded from NMA)** | | | | | | | | | | | |
| Aguilar-Shea 2016 | 68 | NR | NR | NR | NR | NR | NR | NR | NR | NR | NR |
| Ehrlich 2019 | 69 | 54 | 65 | 17 | NR | NR | NR | NR | NR | NR | NR |
| Goehring 2020 | 72 | 55 | 0 | 33 | NR | NR | 43 | NR | 25 | 13 | NR |
| Khachatryan 2021 (EFFECT AF) | 67 | 57 | NR | NR | 70 | 24 | NR | NR | NR | NR | NR |
| La Pointe 2015 | 56 | 66 | 52 | 16 | NR | NR | 23 | NR | NR | NR | NR |
| Malladi 2021 | 64 | 67 | 57 | 10 | 68 | 32 | NR | 16 | 3 | NR | NR |
| Noseworthy 2015 | 60 | 70 | NR | 20 | NR | NR | NR | NR | 21 | 6 | NR |
| Qin 2016 | 70 | 59 | 64 | 21 | 58 | 42 | NR | 36 | 10 | NR | NR |
| Taylor 2010 | NR | 54 | NR | NR | NR | NR | NR | NR | NR | NR | NR |

Abbreviations **–** AF: Atrial fibrillation; CAD: Coronary artery disease; COPD: Chronic obstructive pulmonary disease; HT: Hypertension; LVEF: Left ventricular ejection fraction; MI: Myocardial infarction; NMA: Network meta-analysis; NR: Not reported; RCT: Randomized controlled trial; RWE: Real world evidence; SD: Standard deviation.

**Supplementary Table A.9**. Cochrane risk of bias tool for randomized trials

| **First Author & Year (Study Name)** | **Random Sequence Generation** | **Allocation Concealment** | **Blinding of Participants and Personnel** | **Blinding of Outcome Assessment** | **Incomplete Outcome Data** | **Selective Reporting** | **Other Sources of Bias** |
| --- | --- | --- | --- | --- | --- | --- | --- |
| [AFFIRM First Antiarrhythmic Drug Substudy Investigators](https://pubmed.ncbi.nlm.nih.gov/?sort=date&term=AFFIRM+First+Antiarrhythmic+Drug+Substudy+Investigators%5BCorporate+Author%5D) 2003 (AFFIRM) | Low risk | Low risk | High risk | High risk | Low risk | High risk | Unclear risk |
| Antman 1990 | High risk | High risk | High risk | High risk | Low risk | Low risk | Low risk |
| Bellandi 2001 | Unclear risk | Unclear risk | Unclear risk | Unclear risk | Low risk | Low risk | Unclear risk |
| Benditt 1999 | Low risk | Low risk | Unclear risk | Unclear risk | Low risk | Low risk | Low risk |
| Capucci 2008 (DAPHNE) | Unclear risk | Unclear risk | Unclear risk | Low risk | Low risk | Low risk | Unclear risk |
| de Paola 1999 | Unclear risk | Unclear risk | High risk | High risk | Low risk | High risk | Unclear risk |
| Fetsch 2004 (PAFAC) | Low risk | Low risk | Unclear risk | Unclear risk | Low risk | Low risk | Unclear risk |
| Hohnloser 2009 (ATHENA) | Unclear risk | Unclear risk | Unclear risk | Unclear risk | Low risk | Low risk | Unclear risk |
| Hohnloser 1995 | Unclear risk | Unclear risk | High risk | High risk | Low risk | Low risk | Low risk |
| Juul-Moller 1990 | Unclear risk | Unclear risk | High risk | High risk | Low risk | High risk | Low risk |
| Kochiadakis 2004 | Unclear risk | Unclear risk | Unclear risk | Unclear risk | Low risk | High risk | Low risk |
| Kochiadakis 2000 | Low risk | Low risk | Unclear risk | Unclear risk | Low risk | Low risk | Low risk |
| Kwang Jin 2014 | Unclear risk | Unclear risk | High risk | High risk | Low risk | Low risk | Unclear risk |
| Le Heuzey 2010 (DIONYSOS) | Unclear risk | Unclear risk | Unclear risk | Unclear risk | Low risk | Low risk | Unclear risk |
| Lee 1997 | Unclear risk | Unclear risk | Unclear risk | Unclear risk | High risk | Low risk | Low risk |
| Lombardi 2006 (A-COMET-II) | Low risk | Low risk | Unclear risk | Unclear risk | Low risk | High risk | Unclear risk |
| NCT01182376 (SOAR) | Unclear risk | Unclear risk | Unclear risk | Unclear risk | High risk | Low risk | Low risk |
| NCT01198873 (ODYSSEUS) | Unclear risk | Unclear risk | Unclear risk | Unclear risk | Low risk | Low risk | Low risk |
| Patten 2004 (SOPAT) | Unclear risk | Unclear risk | Unclear risk | Unclear risk | Low risk | Low risk | Unclear risk |
| Plewan 2001 | Unclear risk | Unclear risk | High risk | High risk | Low risk | High risk | Low risk |
| Reiffel 2015 (HARMONY) | Unclear risk | Unclear risk | Unclear risk | Unclear risk | Unclear risk | Low risk | Unclear risk |
| Reimold 1993 | Low risk | Low risk | High risk | High risk | Low risk | Low risk | Low risk |
| Singh 2007 (EURIDIS; ADONIS) | Unclear risk | Unclear risk | Unclear risk | Unclear risk | Low risk | High risk | Low risk |
| Singh 2005 (SAFE-T 2005) | Unclear risk | Unclear risk | Unclear risk | Unclear risk | Low risk | Low risk | Low risk |
| Singh 1991 | Unclear risk | Unclear risk | Unclear risk | Unclear risk | Unclear risk | High risk | Unclear risk |
| Touboul 2003 (DAFNE) | Unclear risk | Unclear risk | Unclear risk | Unclear risk | Low risk | High risk | Unclear risk |
| Vijayalakshmi 2006 | Low risk | Low risk | High risk | High risk | Low risk | High risk | Unclear risk |
| Wanless 1997 | Unclear risk | Unclear risk | Unclear risk | Unclear risk | Low risk | Low risk | Unclear risk |

**Supplementary Table A.10**. Newcastle-Ottawa scale for observational studies

| **First Author & Year (Study name)** | **Selection**  **(maximum = 4)** | **Comparability**  **(maximum = 2)** | **Exposure or Outcome (maximum = 3)** | **Final score**  **(maximum = 9)** |
| --- | --- | --- | --- | --- |
| Aguilar-Shea 2016 | 2 | 0 | 3 | 5 |
| Agusala 2015 | 4 | 2 | 2 | 8 |
| Andersen 2009 | 4 | 1 | 3 | 8 |
| De Vecchis 2019 | 4 | 0 | 3 | 7 |
| Ehrlich 2019 | 4 | 2 | 3 | 9 |
| Friberg 2014 | 4 | 2 | 3 | 9 |
| Friberg 2018 | 4 | 2 | 3 | 9 |
| Gao 2014 | 4 | 2 | 3 | 9 |
| Goehring 2020 | 4 | 2 | 2 | 8 |
| Gwag 2018 | 4 | 2 | 3 | 9 |
| Khachatryan 2021 (EFFECT AF) | 3 | 2 | 2 | 7 |
| Kim 2011 | 4 | 0 | 3 | 7 |
| LaPointe 2015 | 4 | 0 | 2 | 6 |
| Lee 2020 | 4 | 2 | 2 | 8 |
| Malladi 2021 | 3 | 2 | 3 | 8 |
| Mascarenhas 2018 | 3 | 0 | 2 | 5 |
| Noseworthy 2015 | 4 | 2 | 2 | 8 |
| Piccini 2014 | 4 | 2 | 3 | 9 |
| Qin 2016 | 4 | 2 | 3 | 9 |
| Sohns 2014 | 4 | 2 | 2 | 8 |
| Taylor 2010 | 2 | 2 | 3 | 7 |
| Wharton 2020 | 4 | 2 | 2 | 8 |

**Supplementary Table A.11**. NMA analysis of safety and efficacy outcomes

| **Outcomes** | **Analysis** | **Dronedarone Studies: events; patients** | **Sotalol Studies: events; patients** | **Effect measure (95% CrI)** |
| --- | --- | --- | --- | --- |
| **Safety** | | | | |
| All-cause death | All-studies | 7: 296; 19,764 | 18; 8,298; 48,923 | **HR: 0.38 (0.19, 0.74)** |
|  |  |  |  | **RR: 0.32 (0.16, 0.65)** |
|  | RCTs-only | 4: 71; 3,531 | 12: 77; 1,873 | HR: 0.46 (0.21, 1.02) |
|  |  |  |  | **RR: 0.36 (0.15, 0.81)** |
| Cardiovascular death | All-studies | 2: 65; 5,424 | 1: 4; 1,571 | HR: 0.25 (0.04, 1.01) |
|  |  |  |  | RR: 0.18 (0.02, 1.67) |
| Ventricular proarrhythmia | All-studies | 6: 202; 16,574 | 20: 523; 22,824 | RR: 0.33 (0.10, 1.08) |
| Ventricular proarrhythmia | RCTs-only | 3: 9; 3,382 | 13: 34; 1,819 | RR: 0.84 (0.17, 4.17) |
| Conduction disorders | All-studies | 3: 60; 5,766 | 3: 62; 3,528 | RR: 0.49 (0.14, 1.33) |
| **Efficacy** | | | | |
| Cardiovascular hospitalization | All-studies | 3: 1381; 7,239 | 3: 624; 3,455 | RR: 0.79 (0.62, 1.02) |
| Atrial fibrillation hospitalization | All-studies | 2: 570; 4,116 | 1: 273; 1,815 | RR: 0.79 (0.49, 1.25) |
| Heart failure hospitalization | All-studies | 4: 172; 7,279 | 2: 59; 3,386 | RR: 0.76 (0.25, 2.14) |
| Stroke | All-studies | 6: 168; 9,836 | 3: 173; 4,752 | RR: 0.64 (0.40, 1.22) |
| Myocardial infarction | All-studies | 2: 129; 4,938 | 2: 268; 3,386 | RR: 0.55 (0.27, 1.55) |
| Atrial fibrillation recurrence | All-studies | 6: 770; 1,232 | 12: 389; 874 | RR: 1.06 (0.83, 1.34) |
| Atrial fibrillation recurrence | RCTs-only | 4: 726; 1,163 | 10: 358; 771 | RR: 1.10 (0.80, 1.55) |

Abbreviations – CrI: Credible interval; HR: Hazard ratio; RCT: Randomized controlled trial; RR: Risk ratio.

**Bold** values indicate statistical significance.
